# Fine mapping of *Rf5* region for a sorghum fertility restorer gene and microsynteny analysis across grass species

**DOI:** 10.1270/jsbbs.21057

**Published:** 2022-03-10

**Authors:** Atsushi Kiyosawa, Jun-ichi Yonemaru, Hiroshi Mizuno, Hiroyuki Kanamori, Jianzhong Wu, Hiroyuki Kawahigashi, Kazumi Goto

**Affiliations:** 1 Nagano Animal Industry Experiment Station, 10931-1 Kataoka, Shiojiri, Nagano 399-0711, Japan; 2 Institute of Crop Science, National Agriculture and Food Research Organization, 2-1-2 Kannondai, Tsukuba, Ibaraki 305-8602, Japan; 3 Nagano Agricultural Development Public Corporation, Kami-Ina Branch, 3497 Arai, Ina, Nagano 396-8666, Japan

**Keywords:** sorghum, *Rf* (fertility restorer) genes, *Rf5*, fine mapping, microsynteny

## Abstract

Cytoplasmic male sterility (CMS) is widely used to control pollination in the production of commercial F_1_ hybrid seed in sorghum. So far, 6 major fertility restorer genes, *Rf1* to *Rf6*, have been reported in sorghum. Here, we fine-mapped the *Rf5* locus on sorghum chromosome 5 using descendant populations of a ‘Nakei MS-3A’ × ‘JN43’ cross. The *Rf5* locus was narrowed to a 140-kb region in BTx623 genome (161-kb in JN43) with 16 predicted genes, including 6 homologous to the rice fertility restorer *Rf1* (PPR.1 to PPR.6). These 6 homologs have tandem pentatricopeptide repeat (PPR) motifs. Many *Rf* genes encode PPR proteins, which bind RNA transcripts and modulate gene expression at the RNA level. No PPR genes were detected at the *Rf5* locus on the corresponding homologous chromosome of rice, foxtail millet, or maize, so this gene cluster may have originated by chromosome translocation and duplication after the divergence of sorghum from these species. Comparison of the sequences of these genes between fertile and CMS lines identified PPR.4 as the most plausible candidate gene for *Rf5*.

## Introduction

Cytoplasmic male sterility (CMS) is widely used to control pollination in the production of commercial F_1_ hybrid seed, including that of sorghum (*Sorghum bicolor* (L.) Moench). CMS in flowering plants is characterized by a maternally inherited inability to produce functional pollen (Hanson and Bentolila 2004). It is often caused by abnormal transcripts originating from the mitochondrial genome that usually encode chimeric open reading frames (ORFs) containing part of a functional mitochondrial gene (Hanson and Bentolila 2004). The chimeric transcripts may limit the energy supply in mitochondria for pollen formation and result in sterile pollen (Schnable and Wise 1998).

The CMS phenotype is often restored by a fertility restorer (*Rf*) gene through the action of nuclear-derived RNA-binding proteins. In many cases, members of the large family of pentatricopeptide repeat (PPR) proteins (Barkan *et al.* 2012) are encoded by *Rf* genes (Dahan and Mireau 2013). Lines with *Rf* genes that restore CMS in hybrid cultivars are used as pollen donors. The PPR protein targets are primarily abnormal transcripts originating from the mitochondrial genome (reviewed by Barkan and Small 2014, Manna 2015).

PPR proteins can bind RNA transcripts in a sequence-specific modular fashion. Each PPR forms two α-helices, and the series of helix-turn-helix motifs throughout the protein are stacked together to form a superhelix with an RNA-binding groove (Ban *et al.* 2013, Gully *et al.* 2015). The code of recognition between specific amino acids within the PPRs and the target RNA sequence was reported (Barkan *et al.* 2012, Takenaka *et al.* 2013, Yagi *et al.* 2013). The recognition of transcripts is mediated by interactions between the target RNA sequence and the amino acids at positions 4 and 34 of each PPR motif (Barkan *et al.* 2012, Yagi *et al.* 2013). The PPR family in plants has two major subfamilies, called P and PLS. P-subfamily proteins contain a series of 35-amino-acid (aa) PPR motifs (P) and normally lack additional domains (Schmitz-Linneweber and Small 2008). Most of the functionally characterized *Rf* genes are placed in the P subfamily, the members of which induce the cleavage of sterility-associated mitochondrial RNAs in plant mitochondria (Dahan and Mireau 2013). PLS-subfamily proteins contain characteristic triplets of P (35 amino acids), L (long, 35–36 amino acids), and S (short, ~31 amino acids) motifs (Lurin *et al.* 2004). PLS proteins almost always possess C-terminal domains with “E” or “DYW” motifs, and members are thought to function mainly in RNA editing (Lurin *et al.* 2004).

CMS in sorghum was discovered through the interaction of “milo” (A1) cytoplasm with the “kafir” nuclear background (Stephens and Holland 1954). So far, six major fertility restorer genes, *Rf1* to *Rf6*, have been reported in sorghum. High-resolution genetic and physical mapping showed that *Rf1*, on chromosome Chr. 8, encodes a PLS-subfamily PPR protein (Sobic.008G147400 = PPR13) and can restore pollen fertility in A1 cytoplasm (Klein *et al.* 2005). PPR13 contains 14 PPRs as well as a C-terminal E motif (Lurin *et al.* 2004). Comparison of the coding sequences of fertile and sterile plants revealed a non-synonymous substitution (L26 to S27) adjacent to an amino acid insertion (R26) in the protein of sterile plants. A series of single nucleotide polymorphisms and a small insertion/deletion (indel) occur immediately 5ʹ of the PPR13 transcript. The *Rf2* locus was limited to a 236-kbp region of Chr. 2 with 31 predicted ORFs, including a P-subfamily PPR gene with high sequence similarity to rice *Rf1* (Jordan *et al.* 2010). The *Rf2* locus was fine-mapped to a 10.32-kb region on Chr. 2 with only one candidate PPR gene, Sobic.002G057050, by newly developed simple sequence repeat (SSR) markers (Praveen *et al.* 2018), and a potential causative mutation for *Rf2* was identified (Kante *et al.* 2018). *Rf2* can restore pollen fertility in A1 cytoplasm. *Rf3* and *Rf4*, on Chr. 7, restore CMS caused by A3 cytoplasm (Pring *et al.* 1999, Tang *et al.* 1998, Tang and Pring 2003). A3 cytoplasm has a chimeric ORF107 resulting from recombination/duplication with *atp9* in mitochondrial DNA. *Rf3* induces nucleolytic cleavage of ORF107 transcripts. Both *Rf3* and *Rf4* can restore pollen fertility by up to 50% (Kuhlman *et al.* 2006). *Rf5*, on Chr. 5 (Jordan *et al.* 2011), can restore pollen fertility in A1 and A2 cytoplasms. Linkage analyses delimited the *Rf5* locus to a ~584-kb region on Chr. 5 that is predicted to encode 70 genes. Genome informatic analysis identified seven P-subfamily PPR family members in the genomic regions of *Rf5* as candidates of the causal *Rf5* gene (Jordan *et al.* 2011). It was postulated that multiple PPRs at this locus might correspond to active restorer genes. *Rf6*, on Chr. 4, can restore pollen fertility in A1 and A2 cytoplasms (Praveen *et al.* 2015). Linkage analysis delimited the *Rf6* locus to a 43-kb region, where one PPR gene, Sobic.004G004100, occurs among the six predicted genes. Analysis of peptide sequences indicated a frame-shift mutation with a stop codon (TGA) at 286 bp caused by an 11-bp insertion in the sequence of CMS line A127 (Praveen *et al.* 2015).

F_1_ hybrid breeding for sorghum in Japan started in the 1960s. CMS lines introduced from the USA were used as female parents and domestic Japanese lines were used as male parents. F_1_ cultivars with high biomass have been bred for forage use in Japan, but no studies of their restorer genes have been reported. Recently, we reported the mode of inheritance involved in fertility restoration in seven domestic F_1_ cultivars and found that *Rf5* may be involved in the restoration of five of them (Kiyosawa *et al.* 2020). However, its known region is still large at 584 kb (Jordan *et al.* 2011), and fine mapping is needed to develop precise linkage DNA markers. Therefore, here we fine-mapped *Rf5* using the progeny population of F_1_ cultivar ‘Hazuki’ used in a previous study (Kiyosawa *et al.* 2020) and performed nucleotide polymorphism analysis and microsynteny analysis among grass crops to obtain detailed information on *Rf5*.

## Materials and Methods

### Plant materials and phenotyping

The F_1_ hybrid cultivar ‘Hazuki’ (‘Nakei MS-3A’ × ‘JN43’) was self-pollinated to obtain F_4_ and F_6_ populations. F_4_ line F3-78 and F_6_ line F3-78-56-456, with a heterogeneous *Rf5* region, were grown and tested at Nagano Animal Industry Experiment Station, Nagano (137°98ʹE, 36°1ʹN, 771 m a.s.l.), Japan, in 2013 and 2015. We examined fertility restoration and classified individuals with <5% fertility as ‘Sterile’ and those with ≥5% fertility as ‘Fertile’.

### Marker development and fine mapping of *Rf5* region

We used 410 F_4_ plants and 188 F_6_ plants for genetic mapping. We used SSR markers (Yonemaru *et al.* 2009) and indel markers for fine mapping of the *Rf5* region (Supplemental Table 1). Genomic DNA extraction, PCR amplification, and marker genotyping were the same as in our previous study (Kiyosawa *et al.* 2020, Takai *et al.* 2012, Yonemaru *et al.* 2015).

### BAC sequence analysis of *Rf5* region

Bacterial artificial chromosome (BAC) libraries were constructed from young leaves of the CMS maintenance line ‘Nakei MS-3B’ (39 267 clones with an average insert size of 134 kb) and the restorer line ‘JN43’ (30 811 clones, 125 kb). The libraries were prepared by conventional methods, comprising a partial DNA digest with *Hind* III, size fractionation of high-molecular-weight DNA by pulsed-field gel electrophoresis (CHEF, Bio-Rad Laboratories, Hercules, CA, USA), vector ligation (pIndigo BAC-5, Epicentre Biotechnologies, Madison, WI, USA), and transformation into *E. coli* strain DH10B. Positive BAC clones covering the *Rf5* region were screened from each library using tightly linked DNA markers through PCR amplification, and the identified BACs were shotgun-sequenced (Sasaki *et al.* 2002, Wu *et al.* 2003) to provide approximately tenfold sequence coverage.

PCR analysis using PCR markers MS3B_Chr05_2420485 and JN43_Chr05_2589446 (Supplemental Table 1) identified three BAC clones containing inserts from the *Rf5* region: NaMSB-0088G09 from ‘Nakei MS-3B’ and JN43-0015A16 and JN43-0018F16 from ‘JN43’. BAC sequences were deposited in the DDBJ (Acc. Nos. LC494266 and LC494267).

The reference genome sequence used was Sbicolor_v3.0.1_454, derived from ‘BTx623’ (McCormick *et al.* 2018). The structures of the PPR genes were manually corrected in consideration of frameshifts and splicing junctions. As a result, the structures of six PPR genes of ‘BTx623’ used here (PPR.1_B, PPR.2_B, PPR.3_B, PPR.4_B, PPR.5_B, and PPR.6_B) differed from the structures annotated in Sbicolor_v3.1.1_454. The positions and both sets of annotations of the six PPR genes in the *Rf5* region are shown in Supplemental Tables 2 and 3. The mitochondrial genome sequence was derived from ‘BTx623’ (Acc. No. DQ 984518).

### Next-generation DNA sequencing of restorer and CMS lines

To elucidate the relationship between *Rf5* function and sequences, we re-sequenced five restorer lines—‘JN43’, ‘JN290’, ‘SDS7444’, ‘Chohin237.Daikoukaku’, and ‘JN503’—and four CMS lines—‘AMP-21’, ‘Nakei MS-3A’, ‘(954149)A’, and ‘MS175 (932233)A’—used in the five parental combinations to detect a QTL in the *Rf5* region in the F_2_ populations (Kiyosawa *et al.* 2020) by short-read DNB-Seq technology. These data were deposited in the DDBJ Sequence Read Archive under accession number DRA012197. The informatics for the analysis of re-sequencing data was the same as reported previously (Kiyosawa *et al.* 2020) except for reference genome sequence. To obtain the correct alignments in the region rich in structural variation, we merged two sequence datasets aligned by using two sets of reference sequences, Sbicolor_v3.0.1_454 and Sbicolor_v3.0.1_454, with the *Rf5* region replaced by that of ‘JN43’.

### Microsynteny analysis

The genome sequences were obtained from the JGI database (https://genome.jgi.doe.gov/portal/): Osativa_323_v7.0 for rice (Ouyang *et al.* 2007), Sbicolor v3.0.1 for sorghum (McCormick *et al.* 2018), Zmays_493_APGv4 for maize (Schnable *et al.* 2009), and Sitalica v2.2 for foxtail millet (Bennetzen *et al.* 2012). Chromosomal synteny was analyzed in SyMAP v. 5.0 software (Soderlund *et al.* 2006, 2011).

### Phylogenetic analysis

Alignment and phylogenetic reconstructions were performed using the ‘build’ function of ETE3 v. 3.1.1 software (Huerta-Cepas *et al.* 2016) as implemented on the GenomeNet website (https://www.genome.jp/tools-bin/ete/). The maximum likelihood tree was inferred in PhyML v. 20160115 software (parameters --pinv e -f m --nclasses 4 -o tlr --alpha e --bootstrap 100; Guindon *et al.* 2010) and arranged in MEGA7 software (Kumar *et al.* 2016). The amino acid sequences and alignment of PPR proteins are summarized in Supplemental Fig. 1.

## Results

### Mapping of the *Rf5* gene

**Fig. 1.**
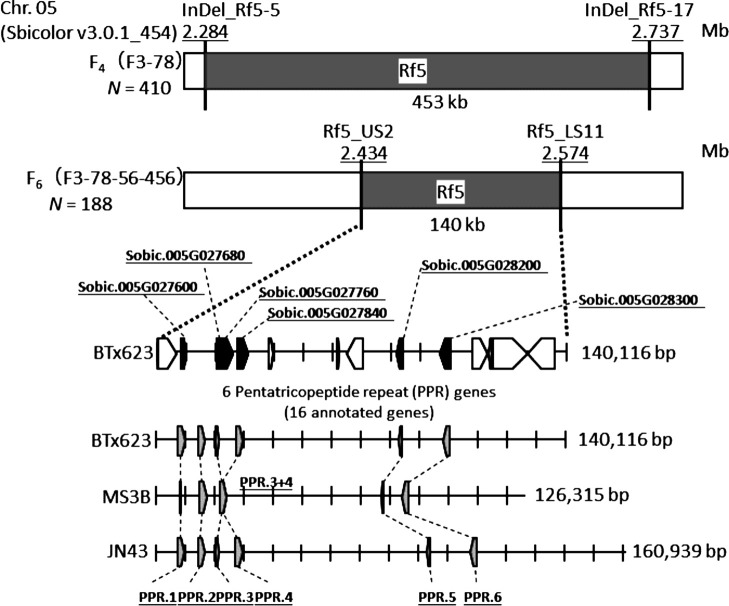
Predicted PPR genes at *Rf5* locus. The *Rf5* locus was mapped to a ~453-kb region on Chr. 5 between markers InDel_Rf5-5 (2.284 Mb) and InDel_Rf5-17 (2.737 Mb) in the F_4_ population (*N* = 410). It was fine-mapped to a ~140-kb region between markers Rf5_US2 (2.434 Mb) and Rf5_LS11 (2.574) in the F_6_ population (*N* = 188). In the target region, 16 genes (black and white boxes) were predicted from the sorghum BTx623 genome (Sbicolor v3.0.1_454), including 6 pentatricopeptide repeat genes in tandem (black boxes). Grey boxes indicate the position and transcription direction of predicted PPR genes at the *Rf5* locus in the total genomic region obtained by the sequencing of BACs of ‘Nakei MS-3B’ and ‘JN43’ and the corresponding region of Chr. 5 of the reference ‘BTx623’ (Sbicolor v3.0.1_454).

To narrow down the *Rf5* region, we examined 410 F_4_ plants. The *Rf5* locus was mapped to the region between markers InDel_Rf5-5 (2.284 Mb) and InDel_Rf5-17 (2.737 Mb) on Chr. 5 (Fig. 1). Further mapping was conducted by screening 188 F_6_ plants (F3-78-56-456), which allowed us to localize *Rf5* to a 140-kb region between markers Rf5_US2 (2.434 Mb) and Rf5_LS11 (2.574 Mb) (Fig. 1). A search of the sorghum genome sequence annotation database v. 3.1 (https://genome.jgi.doe.gov/portal/) revealed 16 predicted genes in this region (Supplemental Table 2), including 6 tandemly repeated PPR genes (PPR.1–PPR.6; Fig. 1, Supplemental Table 3), one or more of which were considered the *Rf5* gene (previously described as 1 of 7 PPR genes in the *Rf5* region, Jordan *et al.* 2011).

### Microsynteny analysis of *Rf5* locus

**Fig. 2.**
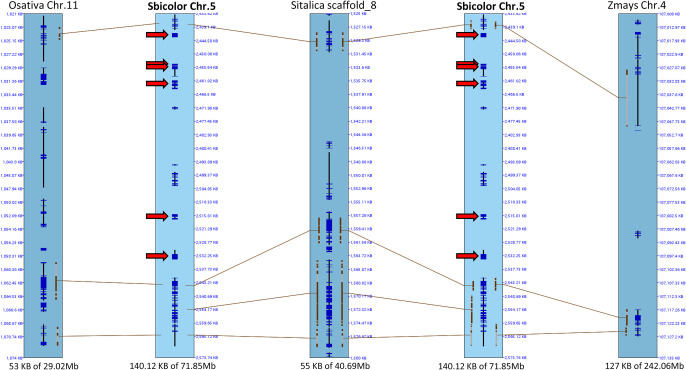
Microsynteny analysis of *Rf5* region. Sorghum Chr. 5 (Sbicolor Chr. 5) has synteny with rice (Osativa) Chr. 11, foxtail millet (Sitalica) scaffold 8, and maize (Zmays) Chr. 4. Six predicted PPR genes (arrows) exist in sorghum but not in the other species.

Comparing the *Rf5* locus of sorghum Chr. 5 (Sbicolor Chr. 05) with homologous chromosomes of rice (Osativa Chr. 11), foxtail millet (Sitalica Scaffold_8), and maize (Zmays Chr. 4), we found no corresponding PPR genes in any of the corresponding chromosomes (Fig. 2). In foxtail millet, homologous PPR genes were located in scaffold 5 (data not shown), not in the corresponding chromosome, scaffold 8. Also, in rice, homologous PPR gene was *Rf4* (Os10g0495200, Kazama and Toriyama 2014) in Chr. 10, not in the corresponding chromosome, Chr. 11.

### Genome sequence analysis

Since the sorghum genome database was constructed from ‘BTx623’, which lacks *Rf5* restoration ability, we isolated and sequenced BAC clones JN43-0015A16 and JN43-0018F16 from a BAC library of the restorer male parent line ‘JN43’ using markers MS3B_Chr05_2420485 (2.425 Mb) and JN43_Chr05_2589446 (2.574 Mb), and BAC clone MS3B-0088G09 from the maintainer line ‘Nakei MS-3B’. Fine mapping defined the length of the *Rf5* locus region as 140 kb in ‘BTx623’, 161 kb in ‘JN43’, and 126 kb in ‘Nakei MS-3B’ (Fig. 1).

In each *Rf5* region, there were 6 PPR genes predicted in ‘BTx623’ and ‘JN43’ and 5 in ‘Nakei MS-3B’, which had a chimeric PPR gene (PPR.3+4) caused by the fusion of the adjacent genes PPR.3 and PPR.4 (Fig. 1). With this exception, a dot plot with genomic sequences (Supplemental Fig. 2) supported no structural indels, translocations, or inversions involving the PPR genes; that is, the PPR genes were conserved among the 3 cultivars.

### PPR.4 of ‘JN43’ is a candidate allele of functional *Rf5*

**Fig. 3.**
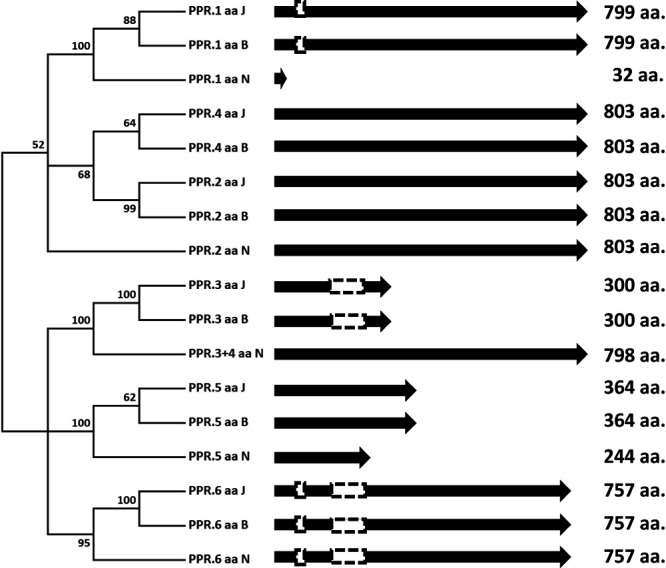
Comparison between PPR proteins. Six PPR genes in the *Rf5* region of ‘BTx623’, ‘JN43’, and ‘Nakei MS-3B’ are compared. Arrows indicate the coding regions of PPR proteins. White boxes indicate deletions in the coding region compared to intact PPR genes assumed. The numbers next to the tree nodes show a bootstrap value (in percent). The number at the right side represents the length of the predicted amino acid sequence.

**Fig. 4.**
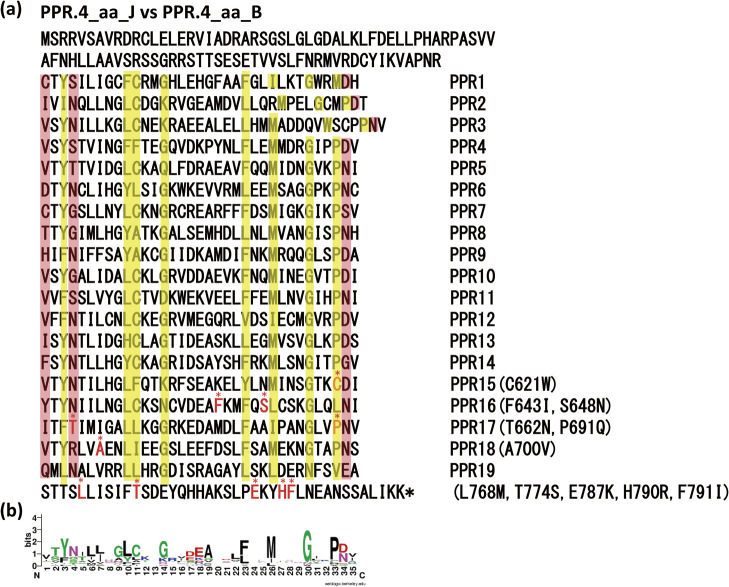
Comparison of amino acid sequences of PPR motifs. (a) Comparison of amino acid sequences of PPR.4_J (left) and PPR.4_B (substitutions on right). Total amino acid sequence of PPR.4_J is shown on the left. The 19 repeats of the PPR motif in units of ~35 aa and their order are listed on the right. Yellow shading indicates amino acids that are relatively conserved in the PPR motif. Pink shading indicates amino acids (1st, 4th, 34th of each motif, except PPR2 and PPR3) important for determining the RNA base recognized by each PPR motif. Red asterisks indicate amino acids that differ between ‘JN43’ (in sequence on left) and ‘BTx623’ (substitution on right). The order of PPR motifs and the positions of amino acid substitutions between ‘JN43’ (left) and ‘BTx623’ (right) are shown on the right. ‘T662N’ in the PPR17 motif is a putative important amino acid substitution for recognizing RNA bases. (b) Consensus sequence of PPR motif predicted from 19 PPR motifs of PPR.4_J and drawn by WebLogo (https://weblogo.berkeley.edu/logo.cgi).

The PPR genes of these 3 cultivars had sequence diversity due to indels, fusions, frame shifts, and gain of a stop codon, and thus their protein lengths varied from 32 to 803 aa (Fig. 3). To distinguish each allele, we appended each gene name with the initial letter of the cultivar name (J = ‘JN43’; B = ‘BTx623’; N = ‘Nakei MS-3B’); for example, PPR.1_B. To identify PPR genes encoding a functional Rf5 protein, we compared amino acid sequences encoded by 6 tandemly repeated PPR genes (PPR.1 to PPR.6) between ‘JN43’ (fertility restorer) and ‘BTx623’ (non-restorer). The sequences of PPR.4_J (fertility restorer), PPR.4_B (non-restorer), and PPR.3+4_N (non-restorer) differed from each other (Table 1). PPR.4_B had 11 aa substitutions compared with PPR.4_J in the PPR motif, among which the T622N substitution may influence the affinity to target RNA (Fig. 4a). There were 127 aa differences between PPR.4_J and PPR.3+4_N (Supplemental Fig. 3); the differences between PPR.4_B and PPR.3+4_N seem to involve a loss of function.

PPR.1 had an I766M substitution between ‘JN43’ and ‘BTx623’ (Supplemental Fig. 1). As the substitution lies in the C-terminal region, which lies outside of the conserved PPR motif, we thought that it does not affect RNA recognition. PPR.3_J encoded 300 aa with only 5 PPR motifs, which suggests that it is not functional as an Rf. Functionally characterized PPR-protein genes for Rf usually contain 11–18 PPR motifs which recognize a specific RNA sequence (Dahan and Mireau 2013). The amino acid sequences of PPR.2, PPR.5, and PPR.6 were identical between ‘JN43’ and ‘BTx623’ (Table 1). These results suggest that PPR.1, PPR.2, PPR.3, PPR.5, and PPR.6 have equivalent functions in ‘JN43’ (restorer) and ‘BTx623’ (non-restorer), and thus are not candidates for the functional *Rf5* gene. Thus, PPR.4 is the most plausible candidate for *Rf5*.

In ‘Nakei MS-3B’ (non-restorer), all PPR genes were drastically changed compared with those in ‘JN43’. PPR.3+4_N had only 84% (676/803) sequence identity with PPR.4_J (Supplemental Fig. 3). The other genes also had lower sequence identity between ‘JN43’ and ‘Nakei MS-3B’ than between ‘JN43’ and ‘BTx623’ (Table 1).

## Discussion

Our previous study showed that *Rf5* is the main restorer gene in Japanese CMS lines (Kiyosawa *et al.* 2020). By detailed mapping of the *Rf5* locus, we delimited it to a 140-kb region of chromosome 5 in BTx623 genome (161-kb in JN43), where six PPR genes were predicted. Since the *Rf5* locus restores the fertility of A1 cytoplasm lines as well as do the *Rf1* and *Rf2* loci, the *Rf5* gene may have high homology with *Rf1* and/or *Rf2*.

Sorghum branched from other Poaceae about 50 million years ago (Gaut 2002). Within the Panicoideae, sorghum branched from millet about 28 million years ago and from maize about 9 million years ago. Microsynteny analysis of the *Rf5* locus detected no PPR genes in rice, foxtail millet, or maize on the corresponding chromosome. These results suggest that the *Rf5* region arose more recently after the branching event from maize, and the PPR genes were translocated from another chromosome and duplicated on Chr. 5.

Nine restorers-of-fertility–like (RFL) PPR genes were reported as a cluster together with other tight clusters containing RFLs on Chr. 5 in sorghum (Sykes *et al.* 2017). This region had 50% (9/18 genes) of the identified RFLs within 554 kb (Sykes *et al.* 2017), which includes the *Rf5* region. This region can be considered a hotspot for recombination at higher rates than expected (Sykes *et al.* 2017), which led to expansion of the family through a probable ‘birth-and death’ process involving diversifying selection (Fujii *et al.* 2011, Geddy and Brown 2007). RFL genes are highly diverse among species and even among strains of the same species, showing strong signals of diversifying selection (Fujii *et al.* 2011, Geddy and Brown 2007). In our study, ‘Nakei MS-3B’ had very different PPR genes in the *Rf5* region from those in the other two cultivars. The coevolution of fertility-restoring PPR genes with CMS-inducing mitochondrial genes has been described as an arms race between the mitochondrial and nuclear genomes (Touzet and Budar 2004). This is similar to the coevolution of plant genes for leucine-rich-repeat–resistance proteins and rapidly evolving pathogen effectors in plant–pathogen interactions (Dahan and Mireau 2013). The difference between ‘Nakei MS-3B’ and the other two cultivars shows the rapid evolution of PPR genes in this region.

### Diversity of PPR motif of sorghum *Rf5* proteins

There were 19 PPR motifs predicted in PPR.4_J (Fig. 4a). The motifs consist of a repetitive sequence of 35 amino acids (36 in PPR2 and 38 in PPR3). In the PPR, amino acids Y3, L10, C11, G14, F23, M26, G30, and P33 are highly conserved (Fig. 4b). These features are consistent with the characteristics of the P-type PPR motifs (Yagi *et al.* 2013). Study of recognition between specific amino acids within the PPRs and target RNA sequences identified the amino acids at position 4 and 34 of each PPR motif as important for the recognition of transcripts, and the amino acid at position 1 restricts the accuracy of the interactions between nucleotides and the protein (Barkan *et al.* 2012, Takenaka *et al.* 2013, Yagi *et al.* 2013). According to the recognition codes of the P-type PPR motifs (Yagi *et al.* 2013), the RNA sequence recognized by the 19 PPR motifs of PPR.4_J was predicted to be 5ʹ-AUCGACAAUGAUUYUCANY-3ʹ.

The sequence of the 17th PPR motif of PPR.4 differed between ‘JN43’ (restorer) and the others (‘BTx623’ and ‘Nakei MS-3B’). In PPR.4_B, the amino acid at position 4 in the 17th PPR motif was changed from T to N, and the conserved 33rd P was changed to Q. In PPR.3+4_N also, the amino acid at position 4 changed from T to N (Supplemental Figs. 1, 3). Thus, both PPR.4_B and PPR.3+4_N had altered PPR motifs, and the nucleotide recognized by these motifs may be important. Two amino acid substitutions in the 17th PPR motif (T to N in the 4th position and P to Q in the 33rd position) perfectly coincide with those in other fertile and CMS lines (Table 2, Supplemental Fig. 4). These data confirm PPR.4 as the most plausible candidate gene for *Rf5*. The change of these PPR motifs may cause the target RNA not to be recognized and result in loss of the restoration of fertility.

In ‘JN43’ with a functional *Rf5* allele, PPR.4 and PPR.2 are paired genes which seem to have been generated by segmental duplication (Fig. 3, Supplemental Fig. 5). PPR.2 also has 19 PPR motifs and seems intact and functional. However, 30 aa substitutions have already occurred between them (Supplemental Fig. 5). The substitutions N128D, N447D, N552D, G622D, D692N (Supplemental Fig. 5) are involved in RNA recognition and suggest these differences in RNA sequence recognition by each PPR protein. Other PPR genes in the *Rf5* region (PPR.1, PPR.3, PPR.5, and PPR.6) have deletions or point mutations making them non-functional (Fig. 3). These results show the rapid evolution of PPR genes in sorghum.

### What is the mitochondrial gene responsible for CMS?

To find genes responsible for CMS, researchers have compared the whole mitochondrial genome between CMS lines and maintainer lines in maize and wheat. Differences of many indels and chimerism of ORFs have been found (Allen *et al.* 2007, Liu *et al.* 2011). However, the causal genes of CMS for *Rf5* in sorghum have not been identified yet. It is important to clarify the sequences of corresponding mitochondrial genes derived from the CMS line that can be rescued by *Rf5*.

Three independent sequences similar to the RNA sequence recognized by the Rf5 protein were found in the mitochondrial genome sequence of ‘BTx623’ (468,628 bp). Two were found in the non-coding region but the other one matched part of the coding region of *rps2b*, which encodes mitochondrial ribosomal protein 2B. Part of the *rps2b* RNA sequence (5ʹ-CAAUGAUUCUCAAT-3ʹ) partially matched 5ʹ-AUCGACAAUGAUUYUCANY-3ʹ, which the PPR.4_J protein recognizes. We consider the mitochondrial *rps2b* gene as a candidate gene for CMS. The Rf5 protein (PPR.4_J) may bind to *rps2b* mRNA, stabilizing it. Interestingly, a maize PPR protein, EMP4 (empty pericarp 4), is necessary to regulate the correct expression of mitochondrial *rps2b* for seed development and plant growth (Gutierrez-Marcos *et al.* 2007). This is an example in which the stabilization of the mRNA of a mitochondrial gene by a PPR protein is required for plant development. Since the mitochondrial genome sequence from sorghum CMS lines has not been obtained yet, we used the whole sorghum mitochondrial sequence from ‘BTx623’. ‘ATx623’ (A line corresponding to ‘BTx623’) may have an indel or chimerization of one or more mitochondrial genes. Detailed comparison of the mitochondrial sequence of an A1 cytoplasmic male sterile line, ‘ATx623’ and its maintainer line ‘BTx623’ may provide information on candidate genes for CMS. Interestingly, *Rf5* restores not only A1 cytoplasmic sterility but also A2 cytoplasmic sterility, and finding mitochondria sequences that interact with the Rf5 protein (PPR.4_J) and determining their relationship will provide important information for F_1_ breeding.

## Author Contribution Statement

AK and JY designed the experiments. AK, JY, HKN, JW, HKW, and KG carried out the experiments. AK, JY, HKW, and HM analyzed the data. JY, HKW, and HM wrote the paper. All authors reviewed and approved the final manuscript.

## Supplementary Material

Supplemental Figures

Supplemental Table 1

Supplemental Table 2

Supplemental Table 3

## Figures and Tables

**Table 1.** Amino acid identity of PPR proteins among ‘BTx623’, ‘Nakei MS-3B’, and ‘JN43’

	‘BTx623’ vs ‘JN43’	‘Nakei MS-3B’ vs ‘JN43’	‘BTx623’ vs ‘Nakei MS-3B’
PPR.1	99.9% (798/799)	3.4% (27/799)	3.4% (27/799)
PPR.2	100% (803/803)	95.6% (768/803)	95.6% (768/803)
PPR.3	99.7% (299/300)	–	–
PPR.4	98.6% (792/803)	–	–
PPR.5	100% (364/364)	63.7% (232/364)*^a^*	63.7% (232/364)*^a^*
PPR.6	100% (757/757)	99.1% (750/757)	99.1% (750/757)

*^a^* The ‘Nakei MS-3B’ sequence has a stop codon in the middle, so the sequence is truncated.

**Table 2.** Variations of amino acids in PPR.4 region between fertile, CMS, and maintainer lines

Position of a.a.	PPR No.	Position of a.a. in each PPR	*Fertile* (*Rf5*)						*CMS* (*rf5*)					Maintainer (*rf5*)
			JN43	JN290	SDS7444	Chohin237. Daikoukaku	JN503		AMP-21	Nakei MS-3A	(954149)A	MS175. (932233)A		BTx623
621	PPR15	33	C	C	C	C	C		W	W	–*^a^*	W		W
643	PPR16	20	F	F	F	F	F		I	I	–	I		I
648	PPR16	25	S	S	S	S	S		N	N	–	N		N
**662** *^b^*	**PPR17**	**4**	**T**	**T**	**T**	**T**	**T**		**N**	**N**	**–**	**N**		**N**
691	PPR17	33	P	P	P	P	P		Q	Q	–	Q		Q
700	PPR18	7	A	A	A	A	A		A	A	–	A		V
768	–	–	L	L	L	L	L		M	M	–	M		M
774	–	–	T	T	T	T	T		S	S	–	S		S
787	–	–	E	E	E	E	E		K	K	–	K		K
790	–	–	H	H	H	H	H		R	R	–	R		R
791	–	–	F	F	F	F	F		I	I	–	I		I

*^a^* Unidentified polymorphisms due to mapping with no or few reads. *^b^* Important amino acid for determining the RNA base recognized by each PPR motif.
