# Supplementary material for: Fine mapping of Rf5 region for a sorghum fertility restorer gene and microsynteny analysis across grass species
Source: Breed Sci. 2022 Mar 10;72(2):141–9. doi: 10.1270/jsbbs.21057 (PMC9522528; doi:10.1270/jsbbs.21057)
Supplement: Supplementary file 1 — Supplemental Figures [file 72_141_s1.pdf]

```
>PPR.1_aa_B
--MSRRVGAAPARCLELERIIAERARSRSLGLDDALKLFDELLHHARPASVRAFNHLLTAVSRARCSSASELAVSHFNRMVRECSDKVAPNLCTYSILIG
RFCRMGHLEHGFAAFGLILKTGWRMDHIAINQLLKGLCHGKRVGEAMDVLLQRMPELGCMPDTVSYTILLKGLCNEKRAEEALELLHMMADDHGRRCPPN
VVSYSIVINGFFTEGQVDKPYNLFLEMIDRGIPPDVVTYTTVIDGLCKAQLFDRAEGVFQQMIDNGFKPNNYTYNCLIHGYLSIGKWKEVVQMLEEMSAR
GLKPDCYTYGSLLNYLCKNGRCREARFFFDSMIRKGIKPKVSTYGILIHGYATKGALSEMHSFLDLMVENGLSPDHHIFNIFFSAYAKCGMIDKAMDIFN
KMRQHGLSPNVVNYGALIDALCKLGRVDDAEVKFNQMINEGVTPNIVVFNSLVYGLCTVDKWERAEELVYEMLDQGICPNAVFFNTLICNLCNVGRVMEG
RRLIDLMEHVGVRPDAFSYTPLISGYCLTGRTDEAEKVFDGMVSIGLSPTEVTYNTLLHGYCSASRIDDAYCLFREMLRKGVTPGVVTYNTILHGLFQTK
RFSEAKELYLNMINSGTKCDIYTYNIILNGLCKSNCVDEAFKMFQSLCSKGLQLNIITFTIMIGALLKGGRKEDAMDLFAAIPANGLVPNVVTYRLVAEN
LIEEGSLEEFDSLFSAMEKNGTAPNSQMLNALVRRLLHRGDISRAGAYLSKLDERNFSVEASTTSLLMSIFTSDEYQHHAKSLPEKYHFLNEANSSALIK
K--------------------------------------------
>PPR.1_aa_J
--MSRRVGAAPARCLELERIIAERARSRSLGLDDALKLFDELLHHARPASVRAFNHLLTAVSRARCSSASELAVSHFNRMVRECSDKVAPNLCTYSILIG
RFCRMGHLEHGFAAFGLILKTGWRMDHIAINQLLKGLCHGKRVGEAMDVLLQRMPELGCMPDTVSYTILLKGLCNEKRAEEALELLHMMADDHGRRCPPN
VVSYSIVINGFFTEGQVDKPYNLFLEMIDRGIPPDVVTYTTVIDGLCKAQLFDRAEGVFQQMIDNGFKPNNYTYNCLIHGYLSIGKWKEVVQMLEEMSAR
GLKPDCYTYGSLLNYLCKNGRCREARFFFDSMIRKGIKPKVSTYGILIHGYATKGALSEMHSFLDLMVENGLSPDHHIFNIFFSAYAKCGMIDKAMDIFN
KMRQHGLSPNVVNYGALIDALCKLGRVDDAEVKFNQMINEGVTPNIVVFNSLVYGLCTVDKWERAEELVYEMLDQGICPNAVFFNTLICNLCNVGRVMEG
RRLIDLMEHVGVRPDAFSYTPLISGYCLTGRTDEAEKVFDGMVSIGLSPTEVTYNTLLHGYCSASRIDDAYCLFREMLRKGVTPGVVTYNTILHGLFQTK
RFSEAKELYLNMINSGTKCDIYTYNIILNGLCKSNCVDEAFKMFQSLCSKGLQLNIITFTIMIGALLKGGRKEDAMDLFAAIPANGLVPNVVTYRLVAEN
LIEEGSLEEFDSLFSAMEKNGTAPNSQMLNALVRRLLHRGDISRAGAYLSKLDERNFSVEASTTSLLISIFTSDEYQHHAKSLPEKYHFLNEANSSALIK
K--------------------------------------------
>PPR.1_aa_N
--MSRRVGAGPARCLELERIIAERDRSRSLGPKP------------------------------------------------------------------
----------------------------------------------------------------------------------------------------
----------------------------------------------------------------------------------------------------
----------------------------------------------------------------------------------------------------
----------------------------------------------------------------------------------------------------
----------------------------------------------------------------------------------------------------
----------------------------------------------------------------------------------------------------
----------------------------------------------------------------------------------------------------
----------------------------------------------------------------------------------------------------
---------------------------------------------
>PPR.2_aa_B
--MSRRVSAVHDRCLELERVIADRARSRSLGLGDALKLFDELLPRARPASVAAFNHLLAAVSR----SSGRRSTTSESETVVSLFNRMVRDCYIKVAPNL
CTYSILIGCFCRMGHLEHGFAAFGLILKSGWRVN-NIVINQLLNGLCDGKRVGEAMDVLLQRMPELGCMPDTVSYNILLKGLCNEKRAEEALELLHMMAD
DQVWSCP-----------------------------------PNVVSYSTVINGFFTEGQVDKPYNLFLEMMDRGIPPDVVTYTTVIDGLCKAQLFDRAE
AVFQQMIDNGVKPNNDTYNCLIHGYLSIGKWKEVVRMLEEMSAGGLKPNCCTYGSLLNYLCKNGRCREARFFFDSMIGKGIKPSVTTYGIMLHGYGNKGA
LSEMHDLLNLMVANGISPNHHIFNIFFSAYAKCGMIDKAMDIFNKMRQHGLSPDAVSYGALIDALCKLGRVDDAEVKFNQMINEGVTPNIVVFSSLVYGL
CSIDKWEKVEELFFEMLNVGIHPNIVFFNTILCNLCKEGRVMEGQRLVDSIECMGVRPDVISYNTLIDGHCLAGTIDEASKLLEGMVSVGLKPNSFSYNT
LLHGYCKAGRIDSAYSLFRKMLSNGITPGVVTYNTILHGLFQTKRFSEAKELYLNMINSGTKWGIYTYNTILNGLCKSNCVDEAFKMFQSLCSKGLQLNI
ITFTIMIGALLKGGRKEDAMDLFAAIPANGLVPDVVTYRVVAENLIEEGSLEEFDSLFSAMEKNGTAPNSQMLNALVRRLLHRGDISRAGAYLSKLDERN
FSVEASTTSMLISIFSSDEYQHHAKSLPKKYRILNEANSSALIKK
>PPR.2_aa_J
--MSRRVSAVHDRCLELERVIADRARSRSLGLGDALKLFDELLPRARPASVAAFNHLLAAVSR----SSGRRSTTSESETVVSLFNRMVRDCYIKVAPNL
CTYSILIGCFCRMGHLEHGFAAFGLILKSGWRVN-NIVINQLLNGLCDGKRVGEAMDVLLQRMPELGCMPDTVSYNILLKGLCNEKRAEEALELLHMMAD
DQVWSCP-----------------------------------PNVVSYSTVINGFFTEGQVDKPYNLFLEMMDRGIPPDVVTYTTVIDGLCKAQLFDRAE
AVFQQMIDNGVKPNNDTYNCLIHGYLSIGKWKEVVRMLEEMSAGGLKPNCCTYGSLLNYLCKNGRCREARFFFDSMIGKGIKPSVTTYGIMLHGYGNKGA
LSEMHDLLNLMVANGISPNHHIFNIFFSAYAKCGMIDKAMDIFNKMRQHGLSPDAVSYGALIDALCKLGRVDDAEVKFNQMINEGVTPNIVVFSSLVYGL
CSIDKWEKVEELFFEMLNVGIHPNIVFFNTILCNLCKEGRVMEGQRLVDSIECMGVRPDVISYNTLIDGHCLAGTIDEASKLLEGMVSVGLKPNSFSYNT
LLHGYCKAGRIDSAYSLFRKMLSNGITPGVVTYNTILHGLFQTKRFSEAKELYLNMINSGTKWGIYTYNTILNGLCKSNCVDEAFKMFQSLCSKGLQLNI
ITFTIMIGALLKGGRKEDAMDLFAAIPANGLVPDVVTYRVVAENLIEEGSLEEFDSLFSAMEKNGTAPNSQMLNALVRRLLHRGDISRAGAYLSKLDERN
FSVEASTTSMLISIFSSDEYQHHAKSLPKKYRILNEANSSALIKK
>PPR.2_aa_N
--MSRRVSAVRDRCLELERVIADRARSGSLGLGDALKLFDELLPHARPASVVAFNHLLAAVSR----SSGRRSTTSESETVVSLFNRMVRDCYIKVAPNR
CTYSILIGCFCRMGHLEHGFAAFGLILKTGWRMD-HIVINQLLNGLCDGKRVGEAMDVLLQRMPELGCMPDTVSYNILLKGLCNEKRAEEALELLHMMAD
DQVWSCP-----------------------------------PNVVSYSTVINGFFTEGQVDKPYNLFLEMIDRGIPPDVVTYTTVIDGLCKAQVVDRAE
GVFQQMIDNGVKPNNDAYNCLIHGYLSIGKWKEVVQMLEEMSARGLKPDCYTYGSLLNYLCKNGRCREARFFFDSMIGKGVKPSVTIYGIMLHGYGNKGA
LSEMHDLLNLMVANGISPSHHIFNIFFSAYAKCGMIDKAMDIFNKMRQQGLSPDAVSYGALIDALCKLGRVDDAEVKFNQMINEGVTPDIVVFSSLVYGL
CTVDKWEKVEELFFEMLNVGIHPNIVFFNTILCNLCKEGRVMEGQRLVDSIECMGVRPDVISYNTLIDGHCLAGTIDEASKLLEGMVSVGLKPDSFSYNT
LLHGYCKAGRIDSAYSHFRKMLSNGITPGVVTYNTILHGLFQTKRFSEAKELYLNMINSGTKWDIYTYNIILNGLCKSNCVDEAIKMFQNLCSKGLQLNI
ITFNIMIGALLKGGRKEDAMDLFAAIPANGLVQNVVTYRLVAENLIEEGSLEEFDSLFSAMEKNGTAPNSQMLNALVRRLLHRGDISRAGAYLSKLDERN
FSVEASTTSMLISIFSSDEYQHHAKSLPKKYRILNEANSSALIKK
```

Supplemental Fig. 1.

Amino acid sequences of PPR proteins used in this study in FASTA format (continued on the next page).

>PPR.4_aa_J
--MSRRVSAVRDRCLELERVIADRARSGSLGLGDALKLFDELLPHARPASVVAFNHLLAAVSR----SSGRRSTTSESETVVSLFNRMVRDCYIKVAPNR
CTYSILIGCFCRMGHLEHGFAAFGLILKTGWRMD-HIVINQLLNGLCDGKRVGEAMDVLLQRMPELGCMPDTVSYNILLKGLCNEKRAEEALELLHMMAD
DQVWSCP-----------------------------------PNVVSYSTVINGFFTEGQVDKPYNLFLEMMDRGIPPDVVTYTTVIDGLCKAQLFDRAE
AVFQQMIDNGVKPNIDTYNCLIHGYLSIGKWKEVVRMLEEMSAGGPKPNCCTYGSLLNYLCKNGRCREARFFFDSMIGKGIKPSVTTYGIMLHGYATKGA
LSEMHDLLNLMVANGISPNHHIFNIFFSAYAKCGIIDKAMDIFNKMRQQGLSPDAVSYGALIDALCKLGRVDDAEVKFNQMINEGVTPDIVVFSSLVYGL
CTVDKWEKVEELFFEMLNVGIHPNIVFFNTILCNLCKEGRVMEGQRLVDSIECMGVRPDVISYNTLIDGHCLAGTIDEASKLLEGMVSVGLKPDSFSYNT
LLHGYCKAGRIDSAYSHFRKMLSNGITPGVVTYNTILHGLFQTKRFSEAKELYLNMINSGTKCDIYTYNIILNGLCKSNCVDEAFKMFQSLCSKGLQLNI
ITFTIMIGALLKGGRKEDAMDLFAAIPANGLVPNVVTYRLVAENLIEEGSLEEFDSLFSAMEKNGTAPNSQMLNALVRRLLHRGDISRAGAYLSKLDERN
FSVEASTTSLLISIFTSDEYQHHAKSLPEKYHFLNEANSSALIKK
>PPR.4_aa_B
--MSRRVSAVRDRCLELERVIADRARSGSLGLGDALKLFDELLPHARPASVVAFNHLLAAVSR----SSGRRSTTSESETVVSLFNRMVRDCYIKVAPNR
CTYSILIGCFCRMGHLEHGFAAFGLILKTGWRMD-HIVINQLLNGLCDGKRVGEAMDVLLQRMPELGCMPDTVSYNILLKGLCNEKRAEEALELLHMMAD
DQVWSCP-----------------------------------PNVVSYSTVINGFFTEGQVDKPYNLFLEMMDRGIPPDVVTYTTVIDGLCKAQLFDRAE
AVFQQMIDNGVKPNIDTYNCLIHGYLSIGKWKEVVRMLEEMSAGGPKPNCCTYGSLLNYLCKNGRCREARFFFDSMIGKGIKPSVTTYGIMLHGYATKGA
LSEMHDLLNLMVANGISPNHHIFNIFFSAYAKCGIIDKAMDIFNKMRQQGLSPDAVSYGALIDALCKLGRVDDAEVKFNQMINEGVTPDIVVFSSLVYGL
CTVDKWEKVEELFFEMLNVGIHPNIVFFNTILCNLCKEGRVMEGQRLVDSIECMGVRPDVISYNTLIDGHCLAGTIDEASKLLEGMVSVGLKPDSFSYNT
LLHGYCKAGRIDSAYSHFRKMLSNGITPGVVTYNTILHGLFQTKRFSEAKELYLNMINSGTKWDIYTYNIILNGLCKSNCVDEAIKMFQNLCSKGLQLNI
ITFNIMIGALLKGGRKEDAMDLFAAIPANGLVQNVVTYRLVVENLIEEGSLEEFDSLFSAMEKNGTAPNSQMLNALVRRLLHRGDISRAGAYLSKLDERN
FSVEASTTSMLISIFSSDEYQHHAKSLPKKYRILNEANSSALIKK
>PPR.6_aa_B
MSRSRRVTAAADRCLELERVIADRAHSGSLGLNDALKLFDEMLTHARPASVRAFNQLLTAVS-----RAR---CSSASELVVSLFNRMIRECSIKVAPSS
FTYTILIGCFCRMGRLKHGFAAFGLILKTGWRVNDTVIFSQLLKGLCDAKRVDEATDILLRRMPEFGCTPDVFSYSILLKGFCNEKRAEEALELLSMMAD
DGDGSHT-----------------------------------PNVVTYTTVIDGLCKAQMVDRAKGVFQHMIDKGVR-----------------------
------------PNNHTYTCLIHGYLSIGKWKEVVQMLQEMSTHGLQPDCYIYAVLLDYLCKNGRCTEARNIFDSVIRKGIKPNVTIYGILLHGYATEGS
LSEMHSFLDLMVGNGVSPDHHIFNIMFCAYAKKAMIDEAMHIFDKMRQQRLSPNIVTYGALIDALCKLGRVDDAVLKFNQMIDEGMTPDIFVFSSLVYGL
CTVDKWEKAEELFFEVLDQGIRLDTVFFNTLMCNLCREGRVMEAQRLIDLMLRVGVRPDVISYNTLVDGHCLTGRIDEAAKLLDVIVSIGLKPDKVTYNT
LLHGYCKARRIDDAYSLFREMLMKGLTPDVVTYNTILHGLFQTGRFSEAKELYLSMINSRTQMNIYTYNIIINGLCKNNFVDEAFKMFHSLCSKDLQLDI
FTANIMIGALLKGGRKEDAMDLFATISAYGLVPDVETYCLIAENLIKEGSLEELDELFSAMEENGTAPNSRMLNALVRWLLHRGDINRAGAYLSKLDEKN
FSLEASTTSMLISIYSRGEYQQLAKSLPEKYHFPQ----------
>PPR.3+4_aa_N
--MPRCVSTVRDRCLELERIIAARARSGSLGLDDALKLFDELLIHARPASVVAFNHILTAVS---RASGRRSSSTSESELVVSLFNRMVRECSIKVAPDT
CTYSILIGCLCRMGRLKHSFATFGLILKTGWRVN-DIVINQLLKGLCDGKRVGEAMDVLLQRMPELGCTPDTVSYNILLKGFCNENRAEEALELLRMMAN
DHGRSCPPDVVSYSTVINGFFTEHQVDKAYNLFLEMMHRGIPPDVATYNTVIDGLCKAQVVDRAEAVFQQMIDNGVKP----------------------
-------------NNDTYNCLIHGYLSIGKWKEVVRMLEEMSAHGPKPDCYTYGPLLNYLCKNGRCREARFFFDSMIGKGVKPSVTIYGIMLHGYGNKGA
LSEMHDLLNLMVANGISPNHHIFNIFFSAYAKCGMIDKAMDIFNKMRQHGLSPNVVNYGALIDALCKLGRVDDAEVKFNQMINEGVTPNIVVFNSLVYGL
CTVDKWERAEELVYEMLDQGICPNAVFFNTLICNLCREGRVMEAQRLIDLMLCVGVRPDVISYNTLVDGHCLTGRIDEAAKLLDVMVSIGLKPDKVTYNT
LLHGYCRARRIDDAYSLFREMLMKGLTPDVVTYNTILHGLFQTGRFSEAKELYLSMINSRTQMNIYTYNIILNGLCKSNCVDEAFKMFQSLCSKGLQLNI
ITFNIMIGALLKGGRKEDAMDLFAAIPANGLVPDVVTYRLVAENLIEEGSLEEFDGLFLTMEKSGTPLDSRLLNALVRRLLQRGDISRAGAYLSKLDEKN
FSLEASTTSELISLFSRGEYQHHAKSLPEKYRLLNEANS------
>PPR.5_aa_N
--MLRRVRAAADRRLQLERFIADRAQSGSLASHDALKLFDLLLPHARPASVTAFNHLLTAVS-----RASGRHSTLESQLGISLFDRMVRECSDKVAPDR
CTYSILIGCFCRMGRLEHGFAVFGLILKTGWRVN-HIVFNQLLKGLCDAKRLDEATNILLWRMPEFGCTPNVVSYNTLVKGFCNENRAEEALELLHVMAD
DQGLSCP-----------------------------------PDVVSYNTVINGFFREGQVDKAYNLFLQMIDRGDSTGCCDIQHSN-------------
----

>PPR.3_aa_J
--MPRCVSTVRDRCLELERIIAARARSGSLGLDDALKLFDELLIHARPASVVAFNQILNAVSRASRASGRRSSSTSESELVVSLFNRMVRECSIKVAPNT
CTYSILIGCLCRMGRLKHSFATFGLILKTGWRVN-DIVINQLLKGLCDGKRVGEAMDVLLQRMPELGCTPDTVSYSILLKGFCNENRAEEALELLRMMAN
DHGRSCP-----------------------------------PNVVTYTTVIDGLCKAQLFDRAEGVFQQMIDNGVKP----------------------
-------------NNDTYNCLIHGYLSIGKWKEVVQMLEEMSARGLKPDCYTYGSLLNYLCKNGRCREARFFF---------------------------
----

Supplemental Fig. 1.

(Continued from previous page, continued on the next page)

>PPR.5_aa_J
--MLRRVRAAADRRLQLERFIADRAQSGSLASHDALKLFDLLLPHARPASVTAFNHLLTAVS-----RASGRHSTLESQLGISLFDRMVRECSDKVAPDR
CTYSILIGCFCRMGRLEHGFAVFGLILKTGWRVN-HIVFNQLLKGLCDAKRLDEATNILLWRMPEFGCTPNVVSYNTLVKGFCNENRAEEALELLHVMAD
DQGLSCP-----------------------------------PDVVSYNTVINGFFREGQVDKAYNLFLQMIDRGIPPDVVTYNTVIDGLCKAQVVDRAK
AVFQQMLDKGVKPSNGTYNCLIHGYLSTGKWKEVVRLLEEMSTHDLEPDCFIYALLLDYLCKNGRCTEARNIFDSVIRKGIKPDVTIYGIMLHGYATEGA
LSEMHEI---

>PPR.5_aa_B
--MLRRVRAAADRRLQLERFIADRAQSGSLASHDALKLFDLLLPHARPASVTAFNHLLTAVS-----RASGRHSTLESQLGISLFDRMVRECSDKVAPDR
CTYSILIGCFCRMGRLEHGFAVFGLILKTGWRVN-HIVFNQLLKGLCDAKRLDEATNILLWRMPEFGCTPNVVSYNTLVKGFCNENRAEEALELLHVMAD
DQGLSCP-----------------------------------PDVVSYNTVINGFFREGQVDKAYNLFLQMIDRGIPPDVVTYNTVIDGLCKAQVVDRAK
AVFQQMLDKGVKPSNGTYNCLIHGYLSTGKWKEVVRLLEEMSTHDLEPDCFIYALLLDYLCKNGRCTEARNIFDSVIRKGIKPDVTIYGIMLHGYATEGA
LSEMHEI---

>PPR.3_aa_B
--MPRCVSTVRDRCLELERIIAARARSGSLGLDDALKLFDELLIHARPASVVAFNQILNAVSRASRASGRRSSSTSESELVVSLFNRMVRECSIKVAPNT
CTYSILIGCLCRMGRLKHSFATFGLILKTGWRVN-DIVINQLLKGLCDGKRVGEAMDVLLQRMPELGCTPDTVSYSILLKGFCNENRAEEALELLRMMAN
DHGRSCP-----------------------------------PNVVTYTTVIDGLCKAQLFDRAEGVFQQMIDNGVKP----------------------
-------------NNDTYNCLIHGYLSIGKWKEVVQMLEKMSARGLKPDCYTYGSLLNYLCKNGRCREARFFF---------------------------
----

>PPR.6_aa_J
MSRSRRVTAAADRCLELERVIADRAHSGSLGLNDALKLFDEMLTHARPASVRAFNQLLTAVS-----RAR---CSSASELVVSLFNRMIRECSIKVAPSS
FTYTILIGCFCRMGRLKHGFAAFGLILKTGWRVNDTVIFSQLLKGLCDAKRVDEATDILLRRMPEFGCTPDVFSYSILLKGFCNEKRAEEALELLSMMAD
DGDGSHT-----------------------------------PNVVTYTTVIDGLCKAQMVDRAKGVFQHMIDKGVR-----------------------
------------PNNHTYTCLIHGYLSIGKWKEVVQMLQEMSTHGLQPDCYIYAVLLDYLCKNGRCTEARNIFDSVIRKGIKPNVTIYGILLHGYATEGS
LSEMHSFLDLMVGNGVSPDHHIFNIMFCAYAKKAMIDEAMHIFDKMRQQRLSPNIVTYGALIDALCKLGRVDDAVLKFNQMIDEGMTPDIFVFSSLVYGL
CTVDKWEKAEELFFEVLDQGIRLDTVFFNTLMCNLCREGRVMEAQRLIDLMLRVGVRPDVISYNTLVDGHCLTGRIDEAAKLLDVIVSIGLKPDKVTYNT
LLHGYCKARRIDDAYSLFREMLMKGLTPDVVTYNTILHGLFQTGRFSEAKELYLSMINSRTQMNIYTYNIIINGLCKNNFVDEAFKMFHSLCSKDLQLDI
FTANIMIGALLKGGRKEDAMDLFATISAYGLVPDVETYCLIAENLIKEGSLEELDELFSAMEENGTAPNSRMLNALVRWLLHRGDINRAGAYLSKLDEKN
FSLEASTTSMLISIYSRGEYQQLAKSLPEKYHFPQ----------
>PPR.6_aa_N
MSRSRRVTAAADRCLELERVIADRAHSGSLGLNDALKLFDEMLTHARPASVRAFNHLLTVVS-----RAR---CSSASELVVSLFNRMIRECSIKVAPSS
FTYTILIGCFCRMGRLKHGFAAFGLILKTGWRVNDTVIFSQLLKGLCDAKRVDEATDILLRRMPEFGCTPDVFSYSILLKGFCNEKRAEEALELLSMMAD
DGDGSHT-----------------------------------PNVVTYTTVIDGLCKAQMVDRAKGVFQHMIDKGVR-----------------------
------------PNNHTYTCLIHGYLSIGKWKEVVQMLQEMSTHGLQPDCYIYAVLLDYLCKNGRCTEARNVFDSVIRKGIKPNVTIYGILLHGYATEGS
LSEMHSFLDLMVGNGVSPDHHIFNIMFCAYAKKAMIDEAMHIFEKMRQQRLSPNIVTYGALIDALCKLGRVDDAVLKFNQMINEGVTPDIFVFSSLVYGL
CTVDKWEKAEELFFEVLDQGIRLDTVFFNTLMCNLCREGRVMEAQRLIDLMLRVGVRPDVISYNTLVDGHCLTGRIDEAAKLLDVMVSIGLKPDKVTYNT
LLHGYCKARRIDDAYSLFREMLMKGLTPDVVTYNTILHGLFQTGRFSEAKELYLSMINSRTQMNIYTYNIIINGLCKNNFVDEAFKMFHSLCSKDLQLDI
FTANIMIGALLKGGRKEDAMDLFATISAYGLVPDVETYCLIAENLIKEGSLEELDELFSAMEENGTAPNSRMLNALVRWLLHRGDINRAGAYLSKLDEKN
FSLEASTTSMLISIYSRGEYQQLAKSLPEKYHFPQ----------

Supplemental Fig. 1. (Continued from previous page)

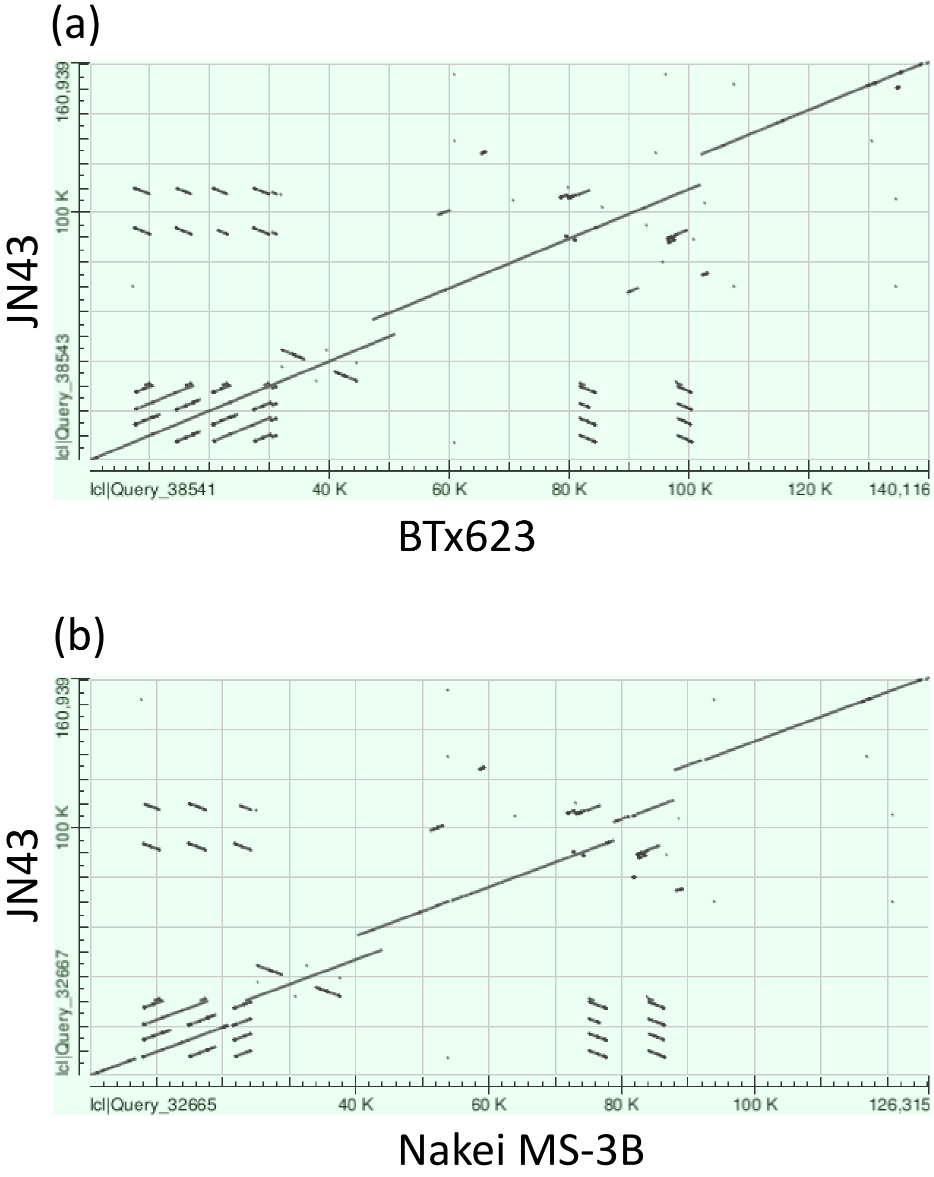

Supplemental Fig. 2.
Dot plots of nucleotide alignments between functional *Rf5* region ('JN43') and non-functional *Rf5* regions (a, 'BTx623'; b, 'Nakei MS-3B').

PPR.4_aa_J vs PPR.3+4_aa_N

MSRRVSAVRDRCLELERVIADRARSGSLGLGDALKLFDELLPHARPASVV
AFNHLLAAVSRSSGRRSTTSESETVVSLFNRMVRDCYIKVAPNR

Sequence	Motif
CTYSILIGCFCRMGHLEHGFAAFGLILKTGWRMDH	PPR1
IVINQLLNGLCDGKRVGEAMDVLLQRMPELGCMPDT	PPR2
VSYNILLKGLCNEKRAEEALELLHMMADDQVWSCPPNV	PPR3
VSYSTVINGFFTEGQVDKPYNLFLEMMDRGIPPDV	PPR4
VTYTTVIDGLCKAQLFDRAEAVFQQMIDNGVKPNI	PPR5
DTYNCLIHGYLSIGKWKEVVRMLEEMSAGGPKPNC	PPR6
CTYGSLLNYLCKNGRCREARFFFDSMIGKGIKPSV	PPR7
TTYGIMLHGYATKGALSEMHDLLNLMVANGISPNH	PPR8
HIFNIFFSAYAKCGIIDKAMDIFNKMRQQGLSPDA	PPR9
VSYGALIDALCKLGRVDDAEVKFNQMINEGVTPDI	PPR10
VVFSSLVYGLCTVDKWEKVEELFFEMLNVGIHPNI	PPR11
VFFNTILCNLCKEGRVMEGQRLVDSIECMGVRPDV	PPR12
ISYNTLIDGHCLAGTIDEASKLLEGMVSVGLKPDS	PPR13
FSYNTLLHGYCKAGRIDSAYSHFRKMLSNGITPGV	PPR14
VTYNTILHGLFQTKRFSEAKELYLNMINSGTKCDI	PPR15
YTYTIILNGLCKSNCVDEAFKMFQSLCSKGLQLNI	PPR16
ITFTIMIGALLKGGRKEDAMDLFAAIPANGLVPNV	PPR17
VTYRLVAENLIEEGSLEEFDSLFSAMEKNGTAPNS	PPR18
QMLNALVRRLLHRGDISRAGAYLSKLDERNFSVEA	PPR19

STTSLLISIFTSDEYQHHAKSLPEKYHFLNEANSSALIKK*

Supplemental Fig. 3. Comparison of PPR motifs of PPR.4_J and PPR.3+4_N. Total amino acid sequence of PPR.4_J is shown on the left. The 19 repeats of the PPR motif in units of ~35 aa and their order are shown on the right. Yellow shading indicates amino acids that are relatively conserved in the PPR motif. Pink shading indicates amino acids (1st, 4th, 34th of each motif) important for determining the RNA base recognized by each PPR motif. Cyan shading indicates amino acids that differ between PPR.4_Jand PPR.3+4_N. Red amino acid residues are critical for RNA recognition and differ between PPR.4_J and PPR.3+4_N.

>PPR.4_aa_JN43_Rf5
MSRRVSAVRDRCLELERVIADRARSGSLGLGDALKLFDELLPHARPASVVAFNHLLAAVSRSSGRRSTTSESETVVSLFNRMVRDCYIKVAPNRCTYSIL
IGCFCRMGHLEHGFAAFGLILKTGWRMDHIVINQLLNGLCDGKRVGEAMDVLLQRMPELGCMPDTVSYNILLKGLCNEKRAEEALELLHMMADDQVWSCP
PNVVSYSTVINGFFTEGQVDKPYNLFLEMMDRGIPPDVVTYTTVIDGLCKAQLFDRAEAVFQQMIDNGVKPNIDTYNCLIHGYLSIGKWKEVVRMLEEMS
AGGPKPNCCTYGSLLNYLCKNGRCREARFFFDSMIGKGIKPSVTTYGIMLHGYATKGALSEMHDLLNLMVANGISPNHHIFNIFFSAYAKCGIIDKAMDI
FNKMRQQGLSPDAVSYGALIDALCKLGRVDDAEVKFNQMINEGVTPDIVVFSSLVYGLCTVDKWEKVEELFFEMLNVGIHPNIVFFNTILCNLCKEGRVM
EGQRLVDSIECMGVRPDVISYNTLIDGHCLAGTIDEASKLLEGMVSVGLKPDSFSYNTLLHGYCKAGRIDSAYSHFRKMLSNGITPGVVTYNTILHGLFQ
TKRFSEAKELYLNMINSGTKCDIYTYNIILNGLCKSNCVDEAFKMFQSLCSKGLQLNIITFTIMIGALLKGGRKEDAMDLFAAIPANGLVPNVVTYRLVA
ENLIEEGSLEEFDSLFSAMEKNGTAPNSQMLNALVRRLLHRGDISRAGAYLSKLDERNFSVEASTTSLLISIFTSDEYQHHAKSLPEKYHFLNEANSSAL
IKK
>PPR.4_aa_JN290_Rf5
MSRRVSAVRDRCLELERVIADRARSGSLGLGDALKLFDELLPHARPASVVAFNHLLAAVSRSSGRRSTTSESETVVSLFNRMVRDCYIKVAPNRCTYSIL
IGCFCRMGHLEHGFAAFGLILKTGWRMDHIVINQLLNGLCDGKRVGEAMDVLLQRMPELGCMPDTVSYNILLKGLCNEKRAEEALELLHMMADDQVWSCP
PNVVSYSTVINGFFTEGQVDKPYNLFLEMMDRGIPPDVVTYTTVIDGLCKAQLFDRAEAVFQQMIDNGVKPNIDTYNCLIHGYLSIGKWKEVVRMLEEMS
AGGPKPNCCTYGSLLNYLCKNGRCREARFFFDSMIGKGIKPSVTTYGIMLHGYATKGALSEMHDLLNLMVANGISPNHHIFNIFFSAYAKCGIIDKAMDI
FNKMRQQGLSPDAVSYGALIDALCKLGRVDDAEVKFNQMINEGVTPDIVVFSSLVYGLCTVDKWEKVEELFFEMLNVGIHPNIVFFNTILCNLCKEGRVM
EGQRLVDSIECMGVRPDVISYNTLIDGHCLAGTIDEASKLLEGMVSVGLKPDSFSYNTLLHGYCKAGRIDSAYSHFRKMLSNGITPGVVTYNTILHGLFQ
TKRFSEAKELYLNMINSGTKCDIYTYNIILNGLCKSNCVDEAFKMFQSLCSKGLQLNIITFTIMIGALLKGGRKEDAMDLFAAIPANGLVPNVVTYRLVA
ENLIEEGSL---RNFSVEASTTSLLISIFTSDEYQHHAKSLPEKYHFLNEANSSAL
IKK
>PPR.4_aa_SDS7444_Rf5
MSRRVSAVRDRCLELERVIADRARSGSLGLGDALKLFDELLPHARPASVVAFNHLLAAVSRSSGRRSTTSESETVVSLFNRMVRDCYIKVAPNRCTYSIL
IGCFCRMGHLEHGFAAFGLILKTGWRMDHIVINQLLNGLCDGKRVGEAMDVLLQRMAELGCMPDTVSYNILLKGLCNEKRAEEALELLHMMADDQVWSCP
PNVVSYSTVINGFFTEGQVDKPYNLFLEMMDRGIPPDVVTYTTVIDGLCKAQLFDRAEAVFQQMIDNGVKPNIDTYNCLIHGYLSIGKWKEVVRMLEEMS
AGGPKPNCCTYGSLLNYLCKNGRCREARFFFDSMIGKGIKPSVTTYGIMLHGYATKGALSEMHDLLNLMVANGISPNHHIFNIFFSAYAKCGIIDKAMDI
FNKMRQQGLSPDAVSYGALIDALCKLGRVDDAEVKFNQMINEGVTPDIVVFSSLVYGLCTVDKWEKVEELFFEMLNVGIHPNIVFFNTILCNLCKEGRVM
EGQRLVDSIECMGVRPDVISYNTLIDGHCLAGTIDEASKLLEGMVSVGLKPDSFSYNTLLHGYCKAGRIDSAYSHFRKMLSNGITPGVVTYNTILHGLFQ
TKRFSEAKELYLNMINSGTKCDIYTYNIILNGLCKSNCVDEAFKMFQSLCSKGLQLNIITFTIMIGALLKGGRKEDAMDLFAAIPANGLVPNVVTYRLVA
ENLIEEGSLEEFDSLFSAMEKNG------------------------GAYLSKLDERNFSVEASTTSLLISIFTSDEYQHHAKSLPEKYHFLNEANSSAL
IKK
>PPR.4_aa_Chohin237.Daikoukaku_Rf5
MSRRVSAVRDRCLELERVIADRARSGSLGLGDALKLFDELLPHARPASVVAFNHLLAAVSRSSGRRSTTSESETVVSLFNRMVRDCYIKVAPNRCTYSIL
IGCFCRMGHLEHGFAAFGLILKTGWRMDHIVINQLLNGLCDGKRVGEAMDVLLQRMPELGCMPDTVSYNILLKGLCNEKRAEEALELLHMMADDQVWSCP
PNVVSYSTVINGFFTEGQVDKPYNLFLEMMDRGIPPDVVTYTTVIDGLCKAQLFDRAEAVFQQMIDNGVKPNIDTYNCLIHGYLSIGKWKEVVRMLEEMS
AGGPKPNCCTYGSLLNYLCKNGRCREARFFFDSMIGKGIKPSVTTYGIMLHGYATKGALSEMHDLLNLMVANGISPNHHIFNIFFSAYAKCGIIDKAMDI
FNKMRQQGLSPDAVSYGALIDALCKLGRVDDAEVKFNQMINEGVTPDIVVFSSLVYGLCTVDKWEKVEELFFEMLNVGIHPNIVFFNTILCNLCKEGRVM
EGQRLVDSIECMGVRPDVISYNTLIDGHCLAGTIDEASKLLEGMVSVGLKPDSFSYNTLLHGYCKAGRIDSAYSHFRKMLSNGITPGVVTYNTILHGLFQ
TKRFSEAKELYLNMINSGTKCDIYTYNIILNGLCKSNCVDEAFKMFQSLCSKGLQLNIITFTIMIGALLKGGRKEDAMDLFAAIPANGLVPNVVTYRLVA
ENLIEE----------------------------VRRLLHRGDISRAGAYLSKLDERNFSVEASTTSLLISIFTSDEYQHHAKSLPEKYHFLNEANSSAL
IKK
>PPR.4_aa_JN503_Rf5
MSRRVSAVRDRCLELERVIADRARSGSLGLGDALKLFDELLPHARPASVVAFNHLLAAVSRSSGRRSTTSESETVVSLFNRMVRDCYIKVAPNRCTYSIL
IGCFCRMGHLEHGFAAFGLILKTGWRMDHIVINQLLNGLCDGKRVGEAMDVLLQRMPELGCMPDTVSYNILLKGLCNEKRAEEALELLHMMADDQVWSCP
PNVVSYSTVINGFFTEGQVDKPYNLFLEMMDRGIPPDVVTYTTVIDGLCKAQLFDRAEAVFQQMIDNGVKPNIDTYNCLIHGYLSIGKWKEVVRMLEEMS
AGGPKPNCCTYGSLLNYLCKNGRCREARFFFDSMIGKGIKPSVTTYGIMLHGYATKGALSEMHDLLNLMVANGISPNHHIFNIFFSAYAKCGIIDKAMDI
FNKMRQQGLSPDAVSYGALIDALCKLGRVDDAEVKFNQMINEGVTPDIVVFSSLVYGLCTVDKWEKVEELFFEMLNVGIHPNIVFFNTILCNLCKEGRVM
EGQRLVDSIECMGVRPDVISYNTLIDGHCLAGTIDEASKLLEGMVSVGLKPDSFSYNTLLHGYCKAGRIDSAYSHFRKMLSNGITPGVVTYNTILHGLFQ
TKRFSEAKELYLNMINSGTKCDIYTYNIILNGLCKSNCVDEAFKMFQSLCSKGLQLNIITFTIMIGALLKGGRKEDAMDLFAAIPANGLVPNVVTYRLVA
ENL--SVEASTTSLLISIFTSDEYQHHAKSLPEKYHFLNEANSSAL
IKK

Supplemental Fig. 4. Amino acid sequences of PPR.4 of both restorer, CMS, and maintainer lines in FASTA format. PPR.4 proteins of five restorer lines—‘JN43’, ‘JN290’, ‘SDS7444’, ‘Chohin237.Daikoukaku’, and ‘JN503’—four CMS lines—‘AMP-21’, ‘Nakei MS-3A’, ‘(954149)A’, and ‘MS175 (932233)A’—, and one maintainer line—‘BTx623’ — (continued on the next page).

```
>PPR.4_aa_AMP-21_Rf5
MSRRVSAVRDRCLELERVIADRARSGSLGLGDALKLFDELLPHARPASVVAFNHLLAAVSRSSGRRSTTSESETVVSLFNRMVRDCYIKVAPNRCTYSIL
IGCFCRMGHLEHGFAAFGLILKTGWRMDHIVINQLLNGLCDGKRVGEAMDVLLQRMPELGCMPDTVSYNILLKGLCNEKRAEEALELLHMMADDQVWSCP
PNVVSYSTVINGFFTEGQVDKPYNLFLEMIDRGIPPDVVTYTTVIDGLCKAQLFDRAEAVFQQMIDNGVKPNIDTYNCLIHGYLSIGKWKEVVRMLEEMS
AGGPKPDCYTYGSLLNYLCKNGRCREARFFFDSMIGKGIKPSVTTYGIMLHGY--KGALSEMHDLLNLMVANGISPSHHIFNIFFSAYAKCGMIDKAMDI
FNKMRQQGLSPDAVSYGALIDALCKLGRVDDAEVKFNQMINEGVTPDIVVFSSLVYGLCTVDKWEKVEELFFEMLNVGIHPNIVFFNTILCNLCKEGRVM
EGQRLVDSIECMGVRPDVISYNTLIDGHCLAGTIDEASKLLEGMVSVGLKPDSFSYNTLLHGYCKAGRIDSAYSHFRKMLSNGITPGVVTYNTILHGLFQ
TKRFSEAKELYLNMINSGTKWDIYTYNIILNGLCKSNCVDEAIKMFQNLCSKGLQLNIITFNIMIGALLKGGRKEDAMDLFAAIPANGLVQNVVTYRLVA
ENLIEEGSLEEFDSLFSAMEKNGTAPNSQMLNALVRRLLHRGDISRAGAYLSKLDERNFSVEASTTSMLISIFSSDEYQHHAKSLPKKYRILNEANSSAL
IKK
>PPR.4_aa_NakeiMS-3A_Rf5
MSRRVSAVRDRCLELERVIADRARSGSLGLGDALKLFDELLPHARPASVVAFNHLLAAVSRSSGRRSTTSESETVVSLFNRMVRDCYIKVAPNRCTYSIL
IGCFCRMGHLEHGFAAFGLILKTGWRMDHIVINQLLNGLCDGKRVGEAMDVLLQRMPELGCMPDTVSYNILLKGLCNEKRAEEALELLHMMADDQVWSCP
PNVVSYSTVINGFFTEGQVDKPYNLFLEMIDRGIPPDVVTYTTVIDGLCKAQLFDRAEAVFQQMIDNGVKPNIDTYNCLIHGYLSIGKWKEVVRMLEEMS
AGGPKPDCYTYGSLLNYLCKNGRCREARFFFDSMIGKGIKPSVTT------GYATKGALSEMHDLLNLMVANGISPSHHIFNIFFSAYAKCGMIDKAMDI
FNKMRQQGLSPDAVSYGALIDALCKLGRVDDAEVKFNQMINEGVTPDIVVFSSLVYGLCTVDKWEKVEELFFEMLNVGIHPNIVFFNTILCNLCKEGRVM
EGQRLVDSIECMGVRPDVISYNTLIDGHCLAGTIDEASKLLEGMVSVGLKPDSFSYNTLLHGYCKAGRIDSAYSHFRKMLSNGITPGVVTYNTILHGLFQ
TKRFSEAKELYLNMINSGTKWDIYTYNIILNGLCKSNCVDEAIKMFQNLCSKGLQLNIITFNIMIGALLKGGRKEDAMDLFAAIPANGLVQNVVTYRLVA
ENLIEEGSLEEFDSLFSAMEKNGTAPNSQMLNALVRRLLHRGDISRAGAYLSKLDERNFSVEASTTSMLISIFSSDEYQHHAKSLPKKYRILNEANSSAL
IKK
>PPR.4_aa_(954149)A_Rf5
----------------------------------------------------------------------------------------------------
-----------------------------------------------------------------------------------------MMADDQVWSCP
PNVVSYSTVINGFFTEGQVDKPYNLFLEMMDRGIPPDVVTYTTVIDGLCKAQLFDRAEAVFQQMIDNGVKPNNDTYNCLIHGYLSIGKWKEVVRMLEEMS
AGGPKPNCCTYGSLLNYLCKNGRCREARFFFDSMIGKGIKPSVTTYGIML--------------------------------------------------
----------------------------------------------------------------------------------------------------
----------------------------------------------------------------------------------------------------
----------------------------------------------------------------------------------------------------
----------------------------------------------------------------------------------------------------
---
>PPR.4_aa_MS175A.(932233)A_Rf5
MSRRVSAVRDRCLELERVIADRARSGSLGLGDALKLFDELLPHARPASVVAFNHLLAAVSRSSGRRSTTSESETVVSLFNRMVRDCYIKVAPNRCTYSIL
IGCFCRMGHLEHGFAAFGLILKTGWRMDHIVINQLLNGLCDGKRVGEAMDVLLQRMPELGCMPDTVSYNILLKGLCNEKRAEEALELLHMMADDQVWSCP
PNVVSYSTVINGFFTEGQVDKPYNLFLEMMDRGIPPDVVTYTTVIDGLCKAQLFDRAEAVFQQMIDNGVKPNIDIYNRLIHGYLSIGKWKEVVRMLEEMS
AGGPKPNCCTYGSLLNYLCKNGRCREARFFFDSMIGKGIKPSVTTYGIMLHGYATKGALSEMHDLLNLMVANGISPNHHIFNIFFRAYAKCGMIDKAMDI
FNKMRQQGLSPDAVSYGALIDALCKLGRVDDAEVKFNQMINEGVTPDIVVFSSLVYGLCTVDKWEKVEELFFEMLNVGIHPNIVFFNTILCNLCKEGRVM
EGQRLVDSIECMGVRPDVISYNTLIDGHCLAGTIDEASKLLEGMVSVGLKPDSFSYNTLLHGYCKAGRIDSAYSHFRKMLSNGITPGVVTYNTILHGLFQ
TKRFSEAKELYLNMINSGTKWDIYTYNIILNGLCKSNCVDEAIKMFQNLCSKGLQLNIITFNIMIGALLKGGRKEDAMDLFAAIPANGLVQNVVTYRLVA
ENLIEEGSLEEFDSLFSAMEKNGTAPNSQMLNALVRRLLHRGDISRAGAYLSKLDERNFSVEASTTSMLISIFSSDEYQHHAKSLPKKYRILNEANSSAL
IKK

>PPR.4_aa_BTx623_Rf5
MSRRVSAVRDRCLELERVIADRARSGSLGLGDALKLFDELLPHARPASVVAFNHLLAAVSRSSGRRSTTSESETVVSLFNRMVRDCYIKVAPNRCTYSIL
IGCFCRMGHLEHGFAAFGLILKTGWRMDHIVINQLLNGLCDGKRVGEAMDVLLQRMPELGCMPDTVSYNILLKGLCNEKRAEEALELLHMMADDQVWSCP
PNVVSYSTVINGFFTEGQVDKPYNLFLEMMDRGIPPDVVTYTTVIDGLCKAQLFDRAEAVFQQMIDNGVKPNIDTYNCLIHGYLSIGKWKEVVRMLEEMS
AGGPKPNCCTYGSLLNYLCKNGRCREARFFFDSMIGKGIKPSVTTYGIMLHGYATKGALSEMHDLLNLMVANGISPNHHIFNIFFSAYAKCGIIDKAMDI
FNKMRQQGLSPDAVSYGALIDALCKLGRVDDAEVKFNQMINEGVTPDIVVFSSLVYGLCTVDKWEKVEELFFEMLNVGIHPNIVFFNTILCNLCKEGRVM
EGQRLVDSIECMGVRPDVISYNTLIDGHCLAGTIDEASKLLEGMVSVGLKPDSFSYNTLLHGYCKAGRIDSAYSHFRKMLSNGITPGVVTYNTILHGLFQ
TKRFSEAKELYLNMINSGTKWDIYTYNIILNGLCKSNCVDEAIKMFQNLCSKGLQLNIITFNIMIGALLKGGRKEDAMDLFAAIPANGLVQNVVTYRLVV
ENLIEEGSLEEFDSLFSAMEKNGTAPNSQMLNALVRRLLHRGDISRAGAYLSKLDERNFSVEASTTSMLISIFSSDEYQHHAKSLPKKYRILNEANSSAL
IKK
```

Supplemental Fig. 4. (Continued from previous page).

PPR.2_aa_J vs PPR.4_aa_J

```
MSRRVSAVHDRCLELERVIADRARSRSLGLGDALKLFDELLPRARPASVA
AFNHLLAAVSRSSGRRSTTSESETVVSLFNRMVRDCYIKVAPNL
CTYSILIGCFCRMGHLEHGFAAFGLILKSGWRVNN        PPR1 (N128D)
IVINQLLNGLCDGKRVGEAMDVLLQRMPELGCMPDT       PPR2
VSYNILLKGLCNEKRAEEALELLHMMADDQVWSCPPNV     PPR3
VSYSTVINGFFTEGQVDKPYNLFLEMMDRGIPPDV        PPR4
VTYTTVIDGLCKAQLFDRAEAVFQQMIDNGVKPNN        PPR5
DTYNCLIHGYLSIGKWKEVVRMLEEMSAGGLKPNC        PPR6
CTYGSLLNYLCKNGRCREARFFFDSMIGKGIKPSV        PPR7
TTYGIMLHGYGNKGALSEMHDLLNLMVANGISPNH        PPR8
HIFNIFFSAYAKCGMIDKAMDIFNKMRQHGLSPDA        PPR9
VSYGALIDALCKLGRVDDAEVKFNQMINEGVTPNI        PPR10 (N447D)
VVFSSLVYGLCSIDKWEKVEELFFEMLNVGIHPNI        PPR11
VFFNTILCNLCKEGRVMEGQRLVDSIECMGVRPDV        PPR12
ISYNTLIDGHCLAGTIDEASKLLEGMVSVGLKPNS        PPR13 (N552D)
FSYNTLLHGYCKAGRIDSAYSLFRKMLSNGITPGV        PPR14
VTYNTILHGLFQTKRFSEAKELYLNMINSGTKWGI        PPR15 (G622D)
YTYNTILNGLCKSNCVDEAFKMFQSLCSKGLQLNI        PPR16
ITFTIMIGALLKGGRKEDAMDLFAAIPANGLVPDV        PPR17 (D692N)
VTYRVVAENLIEEGSLEEFDSLFSAMEKNGTAPNS        PPR18
QMLNALVRRLLHRGDISRAGAYLSKLDERNFSVEA        PPR19
STTSMLISIFSSDEYQHHAKSLPKKYRILNEANSSALIKK*
```

Supplemental Fig. 5. Comparison of PPR motifs of PPR.2_J and PPR.4_J. Total amino acid sequence of PPR.2_J is shown on the left. Cyan shading indicates amino acids that differ between PPR.2_J (in sequence on left) and PPR.4_J (substitution on right). Red amino acid residues are critical for RNA recognition and differ between PPR.2_J and PPR.4_J. Other explanations are as in Fig. S3.
